# Diffuse CO_2_ degassing precursors of the January 2020 eruption of Taal volcano, Philippines

**DOI:** 10.1038/s41598-022-22066-7

**Published:** 2022-11-09

**Authors:** Nemesio M. Pérez, Gladys V. Melián, Pedro A. Hernández, Eleazar Padrón, Germán D. Padilla, Ma. Criselda Baldago, José Barrancos, Fátima Rodríguez, María Asensio-Ramos, Mar Alonso, Carlo Arcilla, Alfredo Mahar Lagmay

**Affiliations:** 1 Instituto Volcanológico de Canarias (INVOLCAN), 38320 San Cristobal de La Laguna, Tenerife, Canary Islands Spain; 2 Instituto Tecnológico y de Energías Renovables (ITER), 38600 Granadilla de Abona, Tenerife, Canary Islands Spain; 3 National Institute of Geological Sciences, University of the Philippines, Diliman, 1101 Quezon City, Metro Manila Philippines; 4 Department of Science and Technology, Philippine Nuclear Research Institute, Quezon City, Philippines

**Keywords:** Geochemistry, Geology, Volcanology

## Abstract

On January 12, 2020, Taal volcano in Philippines erupted, 43 years after its previous eruption in 1977. This eruption was preceded by diffuse CO_2_ degassing precursory signals. Significant temporal variations in diffuse CO_2_ emission from Taal Main Crater Lake (TMLC) were observed across the ~ 12 years reaching high CO_2_ degassing rates in 2011 and 2017, with values typical of plume degassing volcanoes. In addition to these CO_2_ surveys at the TCML, soil CO_2_ efflux continuous monitoring was implemented at Taal volcano since 2016 and a clear increasing trend of the soil CO_2_ efflux in 2017 was observed. These geochemical observations are most simply explained by magma recharge to the system, and represent the earliest warning precursor signals to the January 2020 eruptive activity.

## Introduction

**Figure 1:**
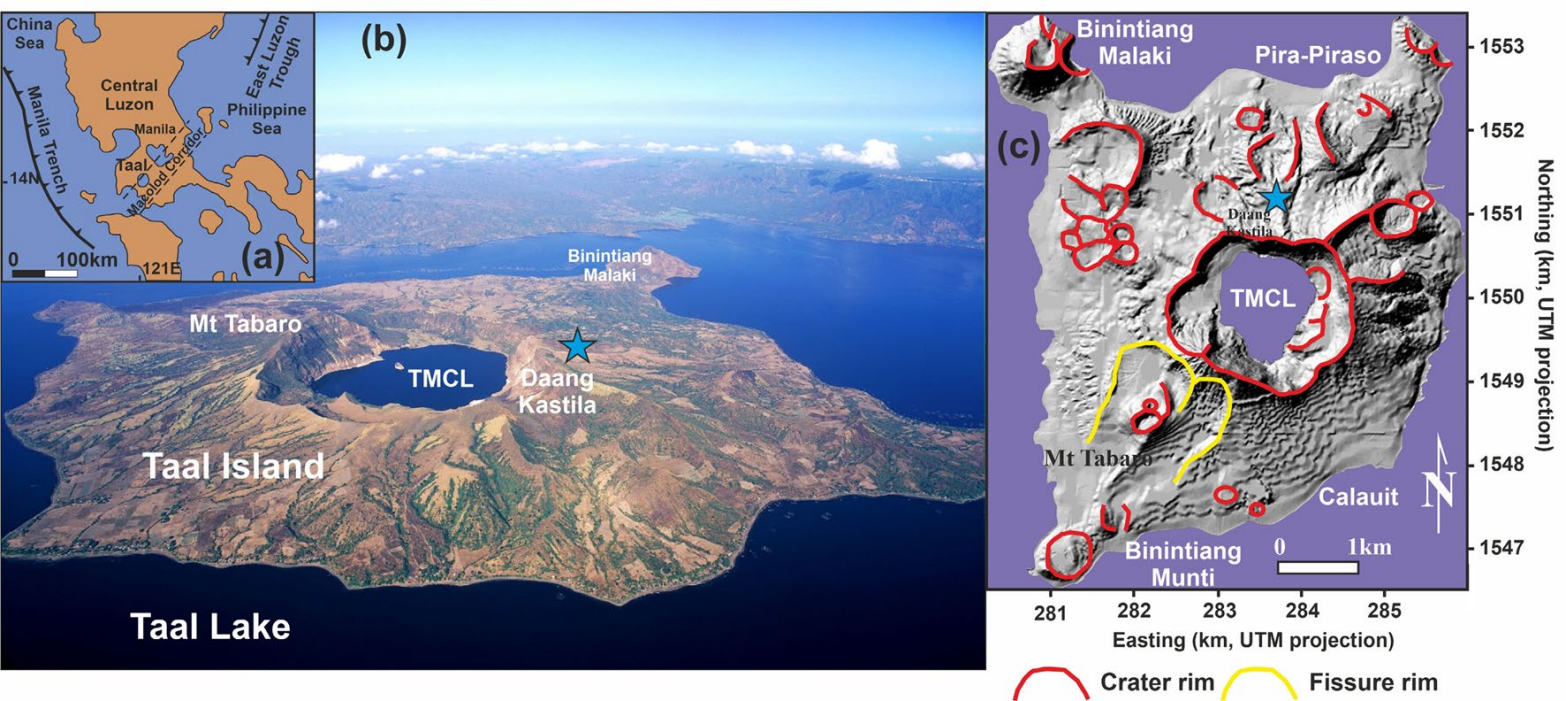
(**a**) Geographical location of Taal Volcano Island, Philippines. (**b**) Aerial view of the volcano taken from the NE showing the TMCL. (**c**) Shaded relief map of Taal Volcano Island showing the Taal Main Crater Lake (TMCL) and the location of the automatic continuous geochemical monitoring station (blue star). The map was constructed with the software Surfer version 8.00 surface mapping system (Golden Software, Inc; https://www.goldensoftware.com/products/surfer).

Taal Volcano Island is the scenario of powerful eruptions and is the largest volcanic threat to the Philippines. The thirty-three recorded eruptions of Taal Volcano Island from 1572 to 1977 include phreatic to phreatomagmatic eruptions. Six of the 33 known eruptions since 1572 have resulted in fatalities^1,2^ and today several million people live within a 20-km radius. Because of these facts, Taal Volcano Island (Fig. 1) was one of the 16 Decade Volcanoes^3^ identified by the International Association of Volcanology and Chemistry of the Earth's Interior (IAVCEI) as worthy of particular study in light of their history of large, destructive eruptions and proximity to populated areas after the United Nations General Assembly designated the 1990s as the International Decade for Natural Disaster Reduction (IDNDR).

Following this international awareness, a collaborative geochemical monitoring research program between Philippine and Spanish scientists was established to contribute to the strengthening of volcanic surveillance of Philippine volcanoes. This collaborative research was focused mainly in the monitoring of diffuse CO_2_ degassing since it is the CO_2_ major gas component, beside water vapor, in both volcanic-hydrothermal fluids and magmas. It is also a good tracer deep of sub-surface magma degassing, since its low solubility in silicate melts at low to moderate pressure favors its early exsolution^4–6^. Volcanogenic CO_2_ is released not only through preferential degassing routes in volcanoes such as fumaroles and plumes, but it could partially also percolate through the entire volcanic edifice and released to the atmosphere in a diffuse or “silent” mode”^7–9^. In addition, large quantities of thermal energy are released by volcanoes through its diffuse CO_2_ emission^10–12^ and this type of degassing can be also a significant contributor to the subaerial global volcanic CO_2_ degassing^13–16^.

Diffuse volcanic degassing disturbs the chemical and isotopic composition of the soil-air and water–air interfaces at the surface environment of the volcano, producing enrichments not only of CO_2_ but also of He, H_2_ and other tracer gases^17–19^. One of the first studies of diffuse degassing on volcanoes was about continuous soil gas H_2_ monitoring at Mount St. Helens^17^. During the last 25 years numerous gas geochemical studies have highlighted the importance of this type of degassing in volcanic systems^7–9,20–26^ and its great use to strengthen the geochemical monitoring program for volcanic surveillance^27–33^, particularly at those volcanic areas where visible volcanic gas emissions (plume, fumaroles, etc.) either are scarce or do not exist^34–36^,. However, the detection of diffuse CO_2_ degassing anomalies prior to volcanic eruptions reported are very scarce^34,37–40^. Hydrothermal fluids, a mixture of seawater, volcanic water and meteoric water^41^, feed the surface discharges of Taal volcano and produced strong hydrothermally altered areas exhibiting solfatara, fumaroles, hot springs, and gas bubbling in the TMCL. Taal volcano has suffered frequent periods of unrest since the eruption in 1977, characterized by increases in the seismic activity, ground deformation and gas emissions. Magmatic intrusions have been the most plausible mechanism to explain these unrests^30,42,43^, although other authors have pointed out that magma intrusions were very unlikely after 1994^44^. On 12 January 2020, a volcanic eruption occurred in the main crater of Taal volcano. The eruption was characterized first by a phreatic-phreatomagmatic style, producing a giant plume of volcanic ash up to ~ 15 km in the atmosphere^45^, and ended with a less explosive eruption characterized by the occurrence of lava fountains. The eruption ejected juvenile products, with evidence of magma mingling^46^ and represented a great impact to the Philippines, as around half a million people were directly affected by the event, producing the loss of ~ 69 M$ worth of damage to infrastructure and agriculture^47^. The acidity of the TMLC waters (pH ~ 3) allows the emission of big amounts of CO_2_ to the atmosphere, as at low pH values, the water of the lake reduces dramatically its ability to dissolve acid gas species as CO_2_. Thus, monitoring CO_2_ emission through the water surface is an important monitoring tool to detect early warning signals of future volcanic unrest episodes. We report here the earliest precursory signal of this volcanic eruption obtained after hundreds of diffuse CO_2_ emission measurements covering the entire surface of the main crater lake and through a continuous monitoring of this gas in a single observation site.

## Results

**Figure 2:**
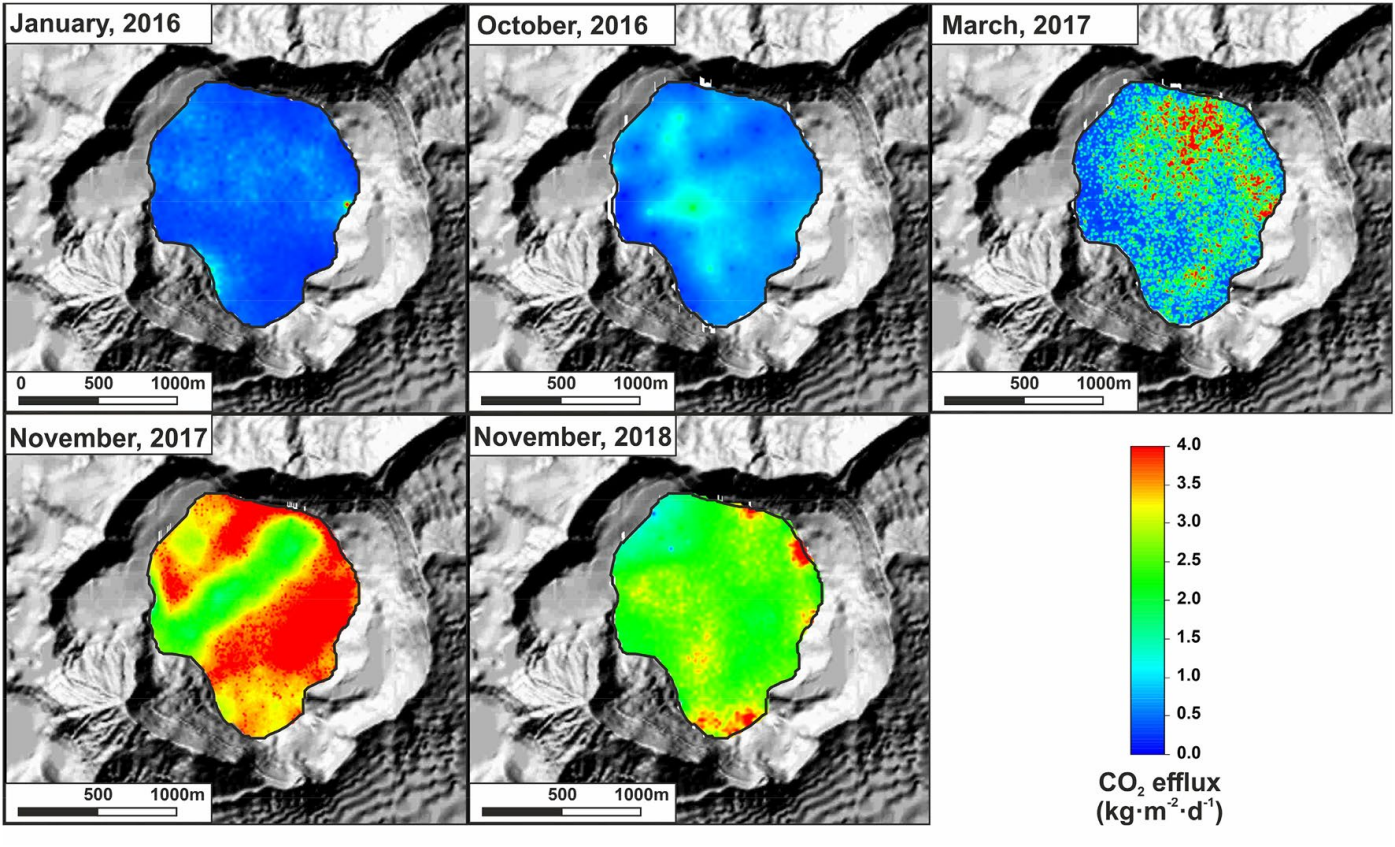
Spatial–temporal variations of CO_2_ efflux measurements at the surface of the TMCL from 2016 to 2018.

About 2630 CO_2_ efflux measurements have been performed covering homogenously the 1.2 km^2^ of the TMCL across the ~ 12 years through 19 surveys (an average of ~ 138 measurements per survey, what means ~ 115 measurements/km^2^) showing a wide range of values from > 0.5 g m^−2^ d^−1^ up to 84,902 g m^−2^ d^−1^. Table 1 summarizes the diffuse CO_2_ emission rate (the amount of CO_2_ that was being released to the atmosphere through the water surface of the TMCL at the time of the survey) observed in the period 2008–2018. Figure 2 shows the spatial distribution of the CO_2_ efflux values in the period 2016–2018, where an important increase of the diffuse CO_2_ emission values can be observed, reaching a relative maximum in November 2017. Statistical-graphical analysis of CO_2_ efflux data of each survey at the TMCL has shown two geochemical populations (background and peak) suggesting the occurrence of either two different CO_2_ sources or degassing dynamics, i.e. advection versus diffusion weight factor on the CO_2_ degassing processes as it has been observed in other volcanic lakes^48^. The background and peak populations of each survey are characterized by relatively low and high CO_2_ efflux values and their average mean values are 643 g m^−2^ d^−1^ and 3707 g m^−2^ d^−1^, respectively.

**Table 1:** Summary of the diffuse CO_2_ emission at TMCL in the period 2008–2018.

Survey date	Diffuse CO_2_ emission TMCL (t d^−1^)	Error (+/−)	Reference
02/04/2008	506	15	Arpa et al.^30^
06/02/2009	948	22	Arpa et al.^30^
01/03/2010	763	18	Arpa et al.^30^
31/08/2010	2716	54	Arpa et al.^30^
08/02/2011	1908	68	Arpa et al.^30^
24/03/2011	4670	159	Arpa et al.^30^
04/05/2011	2057	59	Arpa et al.^30^
25/06/2011	1821	114	Arpa et al.^30^
19/10/2011	482	27	Arpa et al.^30^
07/07/2012	627	22	Arpa et al.^30^
26/10/2013	563	25	This work
26/02/2014	675	36	This work
15/11/2014	2185	124	This work
15/04/2015	1803	160	This work
24/01/2016	532	42	This work
28/10/2016	860	42	This work
16/03/2017	1763	237	This work
16/11/2017	3858	584	This work
22/11/2018	3050	107	This work

**Figure 3:**
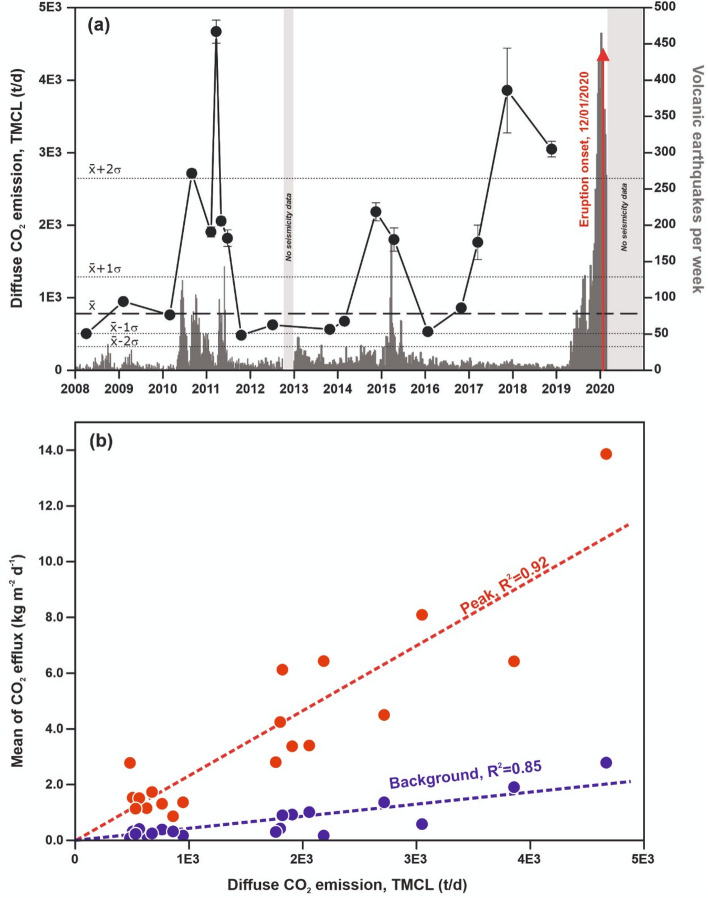
(**a**) Temporal variations of the diffuse CO_2_ degassing rate at the TMCL from 2008 to 2018 (black solid circles and line) and number of volcanic earthquakes per week at Taal Volcano (grey columns) constructed following different published data: from 2008 to 2012^27^ and from 2013 to 2020^39^. (**b**) Correlation between the mean of the two diffuse CO_2_ efflux geochemical populations (background and peak) and the CO_2_ emission rate.

The background diffuse CO_2_ emission from TMCL has been estimated by multiplying the CO_2_ efflux values of the background mean ($\overline{{\text{x}}}$) and range (± 1σ) population times the surveyed area. Therefore, the estimated background diffuse CO_2_ degassing from TMCL shows an average of 782 t d^−1^ with a ± 1σ range values of 1288 and 508 t d^−1^. This background or baseline emission value, established after ~ 12 years of monitoring this geochemical parameter, is in good agreement with the background diffuse CO_2_ emission observed during the period 2008–2010, with a range value between 506 ± 15 and 947 ± 22 t d^−1^. The observed relatively high and anomalous diffuse CO_2_ emission rate along the ~ 12 years reached values of 4670 ± 159 t d^−1^ on March 24, 2011, and 3858 ± 584 t d^−1^ on November 11, 2017, which were higher than $\overline{{\text{x}}}$ + 2σ values (2645 t d^−1^) (Fig. 3a). It is worth noting that the $\overline{{\text{x}}}$ + 1σ value was exceeded in 15 November 2014 and 15 April 2015. Figure 3b depicts the correlation between the mean of the two diffuse CO_2_ efflux geochemical populations (background and peak) and the CO_2_ emission rate and reveals a higher influence of the peak population in the CO_2_ emission rate with its increasing.

**Figure 4:**
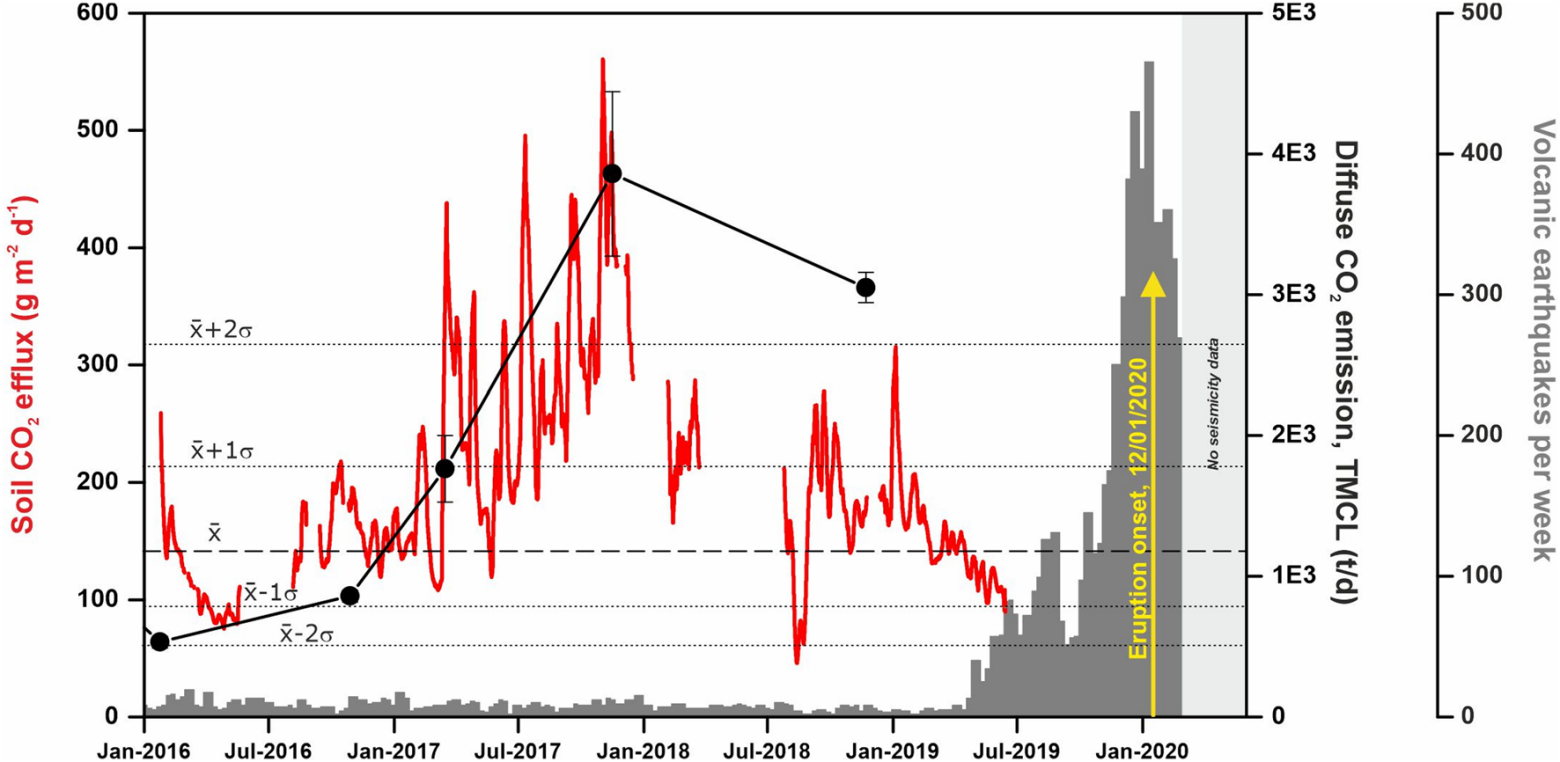
One week moving average of the temporal variations of the soil CO_2_ efflux values measured at the automatic geochemical station (red solid line) and diffuse CO_2_ degassing rate measured at the TMCL (black solid circles and line), in the period January 2016–August 2019. Grey columns show the number of earthquakes per week^39^. Dashed horizontal line depicts the log-normal background population ($\overline{{\text{x}}}$) and dotted horizontal lines depict the background the range ($\overline{{\text{x}}}$ ± 1σ) and ($\overline{{\text{x}}} \pm 2\sigma$) obtained after a probability plot analysis of the soil CO_2_ efflux values measured at the automatic geochemical station.

In order to provide an additional geochemical tool to improve the volcanic surveillance program of Taal, an automatic geochemical station able to measure soil CO_2_ efflux with an hourly frequency, was installed at Daang Kastila (DAK), near the main fissures field on the northern flank of the volcano (Fig. 1). The complete time series measured by the geochemical station is composed of 22,414 valid measurements of soil CO_2_ efflux, wind speed, air humidity and temperature, barometric pressure and soil temperature and moisture, from 25 January 2016 to 31 August 2019. Due to instrumental and power supply problems, time series has a 28.9% of missing data. A probability plot of the data allows us to distinguish two main log-normal geochemical populations: background (70% of the data) and peak (3.8% of the data), with 0.14 kg m^−2^ d^−1^ and 0.50 kg m^−2^ d^−1^ means values, respectively. The average value of the soil CO_2_ efflux data showed oscillations around background values until 14 March 2017. Since that date at 22:00 h, the station measured a sharp increase of soil CO_2_ efflux from ~ 0.1 up to 1.1 kg m^−2^ d^−1^ in 9 h and continued to show a sustained increase in time up to 2.9 kg m^−2^ d^−1^ in 2 November 2017, that represent the main long-term variation of the soil CO_2_ emission time series (Fig. 4). Additionally, the diffuse CO_2_ emission survey carried out on 16 March 2017 at TMCL, showed a relative peak value of 1763 t d^−1^, and was followed by the second maximum value of the diffuse CO_2_ emission survey series (3858 t d^−1^) measured in 16 November 2017, a couple of weeks after the observed maximum value by the automatic geochemical station. One year later, a diffuse CO_2_ emission survey carried out on 22 November 2018 at TMCL, showed a slightly lower value (3050 t d^−1^). The emission rates measured after 2016 were ~ 3.7 times higher in average than the estimated background diffuse CO_2_ emission from TMCL. Similar increases in the CO_2_ released by the TMCL were reported in 2010–2011^30^ and observed in 2014–2015.

## Discussion

The biogenic contribution to the background population at the TMCL seems to be negligible, as the CO_2_ efflux values measured in the Taal Caldera lake reported previously by other authors^49^, where the observed background and peak mean population values (1.3 g m^−2^ d^−1^ and 10.5 g m^−2^ d^−1^, respectively), were two orders of magnitude lower than those observed at the TMCL. Deep-seated CO_2_ degassing has existed from TMCL during the study period and the occurrence of two major populations for the diffuse CO_2_ emission at the TMCL could be closely related to differences of the gas transport mechanism (diffusion and advection) which might be controlled by permeability variations in the volcanic system. The spatial distribution maps of diffuse CO_2_ emission at the TMCL show significant spatial–temporal variations of CO_2_ efflux measurements for the period 2008–2012^30^, and such variations can also be observed for the last 5 surveys performed during the period 2016–2018 (Fig. 2). The observed diffuse CO_2_ emission values from TMLC across the ~ 12 years, reaching high CO_2_ degassing rates in 2011 and 2017, are typical of plume degassing volcanoes^14^ and highly active volcanic systems^14,33^.

The maximum diffuse CO_2_ emission rates across the ~ 12 years (24 March 2011 and 11 November 2017, Fig. 3a) are not only higher than the + 1σ background range values, but also higher than the + 2σ ones (~ 2518 t d^−1^) suggesting higher injection rates of magmatic fluids into the volcanic-hydrothermal system of Taal. The 2010–2011 unrest phase was characterized by significant increases in CO_2_ emission^30^, but the maximum CO_2_ emission rate measured in that period occurred 2 months before the strongest seismic activity recorded during the unrest period. Other episodes of magmatic fluids injection into the volcanic-hydrothermal system likely occurred between 2014 and 2015^43^, and the main increase in seismic activity that generated this magma movement was again preceded by 2–3 months by the increase in CO_2_ emission observed in November 2014. The largest time gap between the observed geochemical anomaly (CO_2_ emission rate) and the seismic activity took place in the third magmatic intrusion that led to the 12 January 2020 eruption, as the maximum CO_2_ emission rate released through the water surface of TMCL occurred in November 2017 or before (Fig. 3a), roughly 17 months before the significant increase recorded in the seismic activity. This increased difference in the time gap between the observed CO_2_ emission rate and the start of anomalous seismic activity suggest a deeper source for the third magmatic intrusion that cause the last CO_2_ emission rate anomaly due to decoupling between the amount of volatile exsolution and magma rise. After November 2017, the temporal variations of the soil CO_2_ efflux values measured at the automatic geochemical station showed an important decrease until it recuperated background values (Fig. 4), likely due to the degassing of the magma that intruded deep at the beginning of 2017.

The inspection of Fig. 3b shows that peak values of the CO_2_ effluxes control in greater proportion the CO_2_ emission rate. Indeed, the slope of the correlation between the mean peak geochemical populations and the CO_2_ emission rate is 5.39 times greater than the background slope. This observation highlight the importance of monitoring the amount of CO_2_ emitted by the lake taking into account the whole surface and not only one observation site, as the selected observation site can be located in an area that exhibits background emission values during the phase of unrest. In fact, and as it is observed in Fig. 2 and has been observed by other authors^30^, the location of CO_2_ emission anomalies varies greatly in TMCL. The highest diffuse emission rates (> 2000 t d^−1^) measured during the study period correspond to the surveys in which the highest peak values were measured (> 3000 g m^−2^ d^−1^), with background emission values < 2000 g m^−2^ d^−1^. This fact can only be explained by the injection of hot magmatic fluids from gas-rich magma into the hydrothermal system and subsequent escape towards the surface through the TMCL.

**Figure 5:**
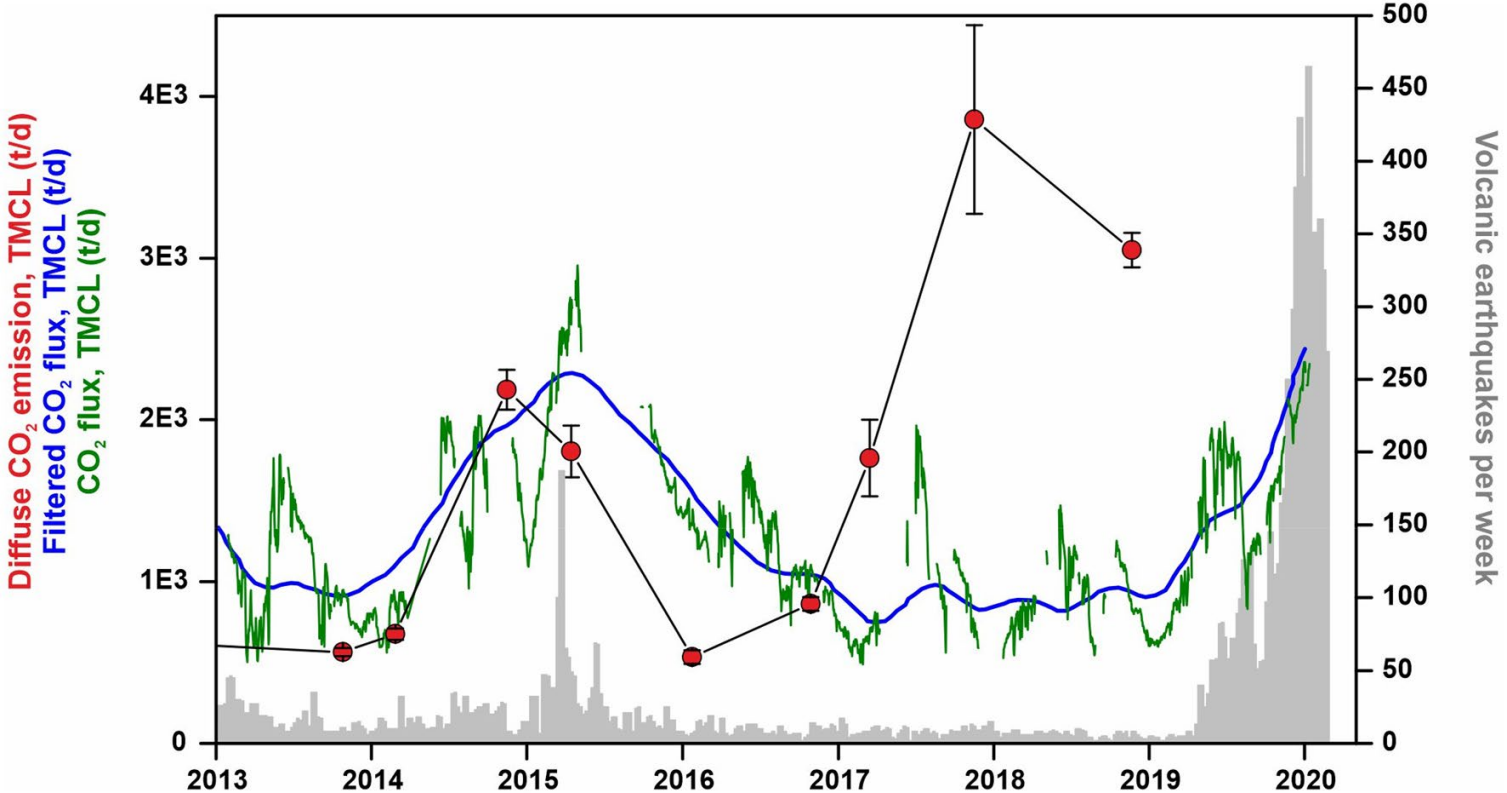
Temporal variations of the diffuse CO_2_ degassing rate at the TMCL from 2013 to 2018 (solid red circles), the CO_2_ flux estimated by the continuous measurement of the partial pressure of CO_2_ dissolved in the lake at a single point of observation^39^ (green line), and the trend of the seasonally adjusted CO_2_ flux data reported by other authors^39^ (blue line). Grey columns show the number of earthquakes per week^39^.

With the aim of quantifying the CO_2_ output released to the atmosphere by TMCL, other authors have proposed an indirect method using continuous measurement of the partial pressure of CO_2_ dissolved in the lake at a single point of observation with a NDIR CO_2_ sensor^43^. Figure 5 shows a comparison between the results obtained by the indirect method and the one showed in this work in the period 2013–2020. Both methods show a relatively good agreement with the exception of the period from August 2017 to the end of the time series: during 2017, the two methods showed an increase in the first half of the year that continued in the data reported in this work (further supported by the data measured at the flank of the volcano by the automatic geochemical station showed in Fig. 4), but is stopped drastically in the emission data obtained by the indirect method^43^ (green line in Fg. 5). Two possible arguments or a combination of both can be made to explain this lack of correlation between both measurement methods in the second half of 2017: (1) the indirect method shows a systematic increase in the estimated CO_2_ emission rate during the dry season of Taal (from November to June), and a systematic decrease in the rainy seasons, that might be caused by the so-called gas *beracun*^50,51^; the process is driven by an stratification in acidic volcanic lakes caused by the entering of cold and fresh rainwater that coated the acidic lake waters with less saline, less acidic, and colder meteoric water. This process would lead a less efficient flushing of CO_2_ through the lake’s top^50,51^ and a worse or less accurate response of the indirect method to estimate the CO_2_ emission rate from the entire surface of TMCL during the rainy season, from June to October; and (2) the loss of the correlation between the pCO_2_ at the measuring site and the CO_2_ emission rate of the TMCL after 2016, because, as was mentioned before, the measuring site might be located in an area that exhibits background emission values during the phase of unrest.

The direct method to estimate the CO_2_ emission rate from the TMCL used in this work, was able to detect a significant increase in 2017–2018 that represents the main and earliest precursor sign of the January 2020 eruption. The robustness of the 2017–2018 precursory increase of the diffuse CO_2_ emission is supported also by the coeval increase observed in the automatic geochemical station (Fig. 4). The soil CO_2_ efflux values measured at the automatic geochemical station suggests that the volatiles exsolved in the third magmatic intrusion likely started to reach the surface of the volcano on March 2017, but strain and stress changes in the crust due to such magma rise at depth were able to produce a significant increase of the seismic activity roughly 17 months after (Fig. 5). Other pulses of CO_2_ emission cannot be ruled out in Taal due to subsequent magma movements due to the absence of diffuse CO_2_ emission data after August 2019. The excellent agreement between the CO_2_ emission values measured at TMCL and the soil CO_2_ efflux values measured at the automatic geochemical station (Fig. 4), confirms that the latter methodology is an excellent complement to the gas emission surveys.

The gas emission data reported here, monitored with much higher detail since January 2016, have been very useful to provide a more reliable long-term warning of the 2020 eruption compared to the onset of precursory seismicity. The inspection of Fig. 3a suggests that between 2010 and 2012, magma rose to shallower crustal positions beneath Taal and that this upward magma movement was accompanied by a remarkable seismic unrest. Later, in 2014, degassing preceded the seismic unrest in few months. This observation might be due to a new slow and aseismic intrusion of magma in a shallower level that later exceeded the strength of the shallow host rocks^52^, or that the observed 2015 seismic unrest was triggered by the quiescent degassing caused by vesiculation or crystallization of the stagnant magma at shallower depths as suggested by other authors in different volcanic systems^53^. During the third magmatic intrusion that led to the volcanic eruption, its initial phase started likely in March 2017 or before and was almost aseismic probably because it occurred at deeper levels and/or magma utilized existing open conduits produced by the two previous unrest stages. The process continued until November 2017, and later magma upward movements (until January 2020), were progressively causing the destabilization of the stagnant magma and opened the eruptive conduit, accompanied by the most energetic seismic activity of the period 2008–2020. Other geochemical parameters seem to support the occurrence of an injection of magmatic fluids into the Taal volcano hydrothermal system from the rise of fresh less and-degassed magma in 2017–2018^54^. The temporal variations of the soil CO_2_ efflux values measured at the automatic geochemical station preceded changes in the seismicity recorded by the Philippine Institute of Volcanology and Seismology (PHIVOLCS), similarly to what has been observed in other volcanic systems^55^, and showed an excellent agreement with the discrete surveys performed in the same period (Fig. 4). This observation confirms the utility of the continuous monitoring of gas emission to monitor more accurately the timing of magma movements at depth.

## Conclusions

The 10 years series of diffuse CO_2_ degassing rate form TMCL, completed since 2016 with a continuous time series of soil CO_2_ efflux in the north flank of the volcano, have been very useful to detect three early precursory signals of magmatic intrusion occurring beneath Taal volcano. Such magmatic rise led anomalous increase of the CO_2_ emission rate from the TMCL measured in 2010–2011 and 2015 that preceded important changes in the seismic activity and other geophysical parameters of roughly 2–3 month, likely due to the decoupling between the amount of CO_2_ exsolved from the ascending magma and the levels of the magmatic rise. New injection of magmatic volatiles into the hydrothermal system in 2017–2018 was caused by a third magmatic rise that likely occurred at deeper zones and could have been enough to trigger the 2020 volcanic eruption.

The geochemical data presented in this study represent the earliest warning precursor signals to the January 2020 eruptive activity. Both discrete CO_2_ emission surveys and the continuous soil CO_2_ efflux monitoring performed at a specific location have provided useful information to detect magma rise episodes. Continuous CO_2_ flux monitoring definitely helps to accurately forecast volcanic eruptions, but regular CO_2_ emission surveys should be promoted in order to complete the information as they cover wider areas of the volcano.

## Methods

The statistical-graphical analysis of the data was based on representing the cumulative normal distribution of the data on a probability scale^56^. On this scale, if a distribution is normal, the plot of the cumulative normal distribution versus the values results in a straight line. The direct reading of the variable at 50% probability provides the value of the average value ($\overline{{\text{x}}}$); the 84% probability reading provides the average value plus one standard deviation ($\overline{{\text{x}}} + \sigma$) and at 16% the average value minus one standard deviation ($\overline{{\text{x}}} - \sigma$). Similarly, $\overline{{\text{x}}} + 2\sigma$ and $\overline{{\text{x}}} - 2\sigma$ can be read at the 2% and 98% percentiles.

Diffuse CO_2_ emission surveys from the TMCL (1.216 km^2^) were carried out performing an average 140 surface CO_2_ efflux measurements per survey at the water–air interface and following the accumulation chamber method^57^. The CO_2_ efflux-meter was equipped with an non-dispersive IR CO_2_sensor LICOR LI-820, able to measure in the range 0–2 mol%, with an accuracy of ~ 4%. The accumulation chamber connected to the non-dispersive IR CO_2_ sensor was mounted on a floating device to allow the measurement at the water surface^25^. The reproducibility of the CO_2_ efflux-meter is 10% in the range 100−10,000 g m^–2^ d^–1^. This random error is based on the uncertainty calculated from the variability of the measurements carried out in the laboratory. In order to convert volumetric to mass flux rates, atmospheric pressure, temperature, and height of the accumulation chamber were taken into account. CO_2_ efflux spatial distribution maps were constructed using conditional sequential Gaussian simulations. 100 equiprobable simulations were made by means of sGs algorithm^58,59^, according to an experimental variogram model that fitted the experimental variogram. Each map is constructed by 3,041 square interpolated cells of 400 m^2^ of surface and the CO_2_ emission rate was estimated by the sum of the cells of the 100 simulations average map. The standard deviations of the 100 simulated values of total CO_2_ output were assumed to be the characteristic values of its uncertainty^59^. Continuous monitoring of soil CO_2_ efflux measurements were performed by an automatic geochemical station installed in the northern sector of Taal volcano (14°1′14.3"N, 120°59′56.6"E) on 25 January 2016. A mechanical arm automatically placed a chamber over the ground every hour, and the soil CO_2_ efflux was measured according to the accumulation chamber method^57^ and an infrared sensor DRÄGER POLYTRON IR CO_2_. Additionally, to avoid a possible influence of external parameters in the endogenous CO_2_ emissions, soil water content and temperature at a depth of 40 cm and atmospheric parameters (wind speed and direction, air temperature and humidity, rainfall, and barometric pressure 1 m above the ground) were recorded simultaneously. All data were stored on an SD memory card and sent by GSM telemetry to the ITER-INVOLCAN facilities.

## Data Availability

All data generated or analyzed during this study are included in this published article. The complete diffuse CO_2_ efflux time series measured by the geochemical station at Taal volcano, can be found at: https://zenodo.org/record/6627433#.YqmWFqGZO5c.
